# Supplementary material for: Causal Relationship between Aging and Anorexia Nervosa: A White-Matter-Microstructure-Mediated Mendelian Randomization Analysis
Source: Biomedicines. 2024 Aug 16;12(8):1874. doi: 10.3390/biomedicines12081874 (PMC11351342; doi:10.3390/biomedicines12081874)
Supplement: Supplementary file 1 [file biomedicines-12-01874-s001.zip › Supplementary Figure/Figure S8/Leaveoneout plot of MR analysis for TL to ubm-b-2016.pdf]

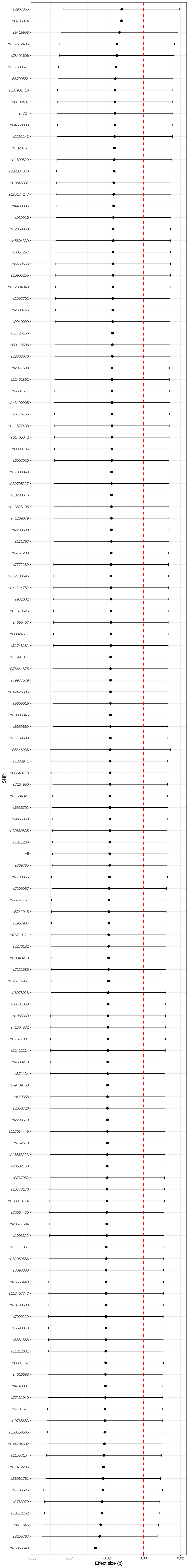
rs2967355
rs3785074
rs9419958
rs117512405
rs78491606
rs117630647
rs56799554
rs137901416
rs8102497
rs4724
rs34550383
rs1291143
rs1023767
rs11699829
rs202034370
rs10845387
rs185174247
rs4498805
rs939916
rs12369950
rs45604339
rs6054257
rs6669563
rs10905255
rs112394943
rs1907702
rs2538745
rs9940099
rs11426156
rs80116508
rs28363070
rs2977608
rs12451892
rs6587577
rs143190905
rs6776756
rs111527438
rs61405042
rs9398196
rs6007020
rs17803849
rs139795227
rs13230646
rs113525195
rs41269079
rs2230590
rs131797
rs6751209
rs7772289
rs142730696
rs141214782
rs932002
rs11579626
rs4695407
rs80324517
rs61748181
rs11991877
rs376641875
rs79977579
rs142426306
rs9600019
rs10805346
rs6659669
rs11769630
rs35446936
rs1332941
rs35640778
rs7164950
rs11584821
rs6536702
rs9955360
rs139669835
rs1611236
All
rs869785
rs7790856
rs7209057
rs55747751
rs5742915
rs1957937
rs76219171
rs2276182
rs10840270
rs7221585
rs145114957
rs16978028
rs66731853
rs1985369
rs41304832
rs17677991
rs12412214
rs4530278
rs871134
rs59409453
rs429358
rs2056726
rs2293579
rs117034449
rs762679
rs116863223
rs28502153
rs3767952
rs10773176
rs188918174
rs75664430
rs28577594
rs1003322
rs11117354
rs182059586
rs3093888
rs76666449
rs117407747
rs73730598
rs7099229
rs6590343
rs6881568
rs11212631
rs3891167
rs4616688
rs4743037
rs77231040
rs4731541
rs10768683
rs150150565
rs144204502
rs11557154
rs11412296
rs56061761
rs7705526
rs2763979
rs10112752
rs611646
rs8105767
rs76065543
SNP
-0.06
-0.04
-0.02
0.00
0.02
Effect size (b)
